# Pulmonary Protection Strategies in Cardiac Surgery: Are We Making Any Progress?

**DOI:** 10.1155/2015/416235

**Published:** 2015-10-20

**Authors:** Emad Al Jaaly, Mustafa Zakkar, Francesca Fiorentino, Gianni D. Angelini

**Affiliations:** ^1^Bristol Heart Institute, Bristol Royal Infirmary, Upper Maudlin Street, Bristol BS2 8HW, UK; ^2^Cardiac Surgery, National Heart and Lung Institute, Imperial College London, Hammersmith Hospital Campus, Du Cane Road, London W12 0HS, UK

## Abstract

Pulmonary dysfunction is a common complication of cardiac surgery. The mechanisms involved in the development of pulmonary dysfunction are multifactorial and can be related to the activation of inflammatory and oxidative stress pathways. Clinical manifestation varies from mild atelectasis to severe respiratory failure. Managing pulmonary dysfunction postcardiac surgery is a multistep process that starts before surgery and continues during both the operative and postoperative phases. Different pulmonary protection strategies have evolved over the years; however, the wide acceptance and clinical application of such techniques remain hindered by the poor level of evidence or the sample size of the studies. A better understanding of available modalities and/or combinations can result in the development of customised strategies for the different cohorts of patients with the potential to hence maximise patients and institutes benefits.

## 1. Introduction

Pulmonary dysfunction is a common complication of cardiac surgery that can impact patient's outcomes and health economics. It is recognised that many patients will have altered pulmonary mechanics after surgery which may appear in a wide range of clinical presentations, from mild atelectasis to life threatening acute lung injury (ALI) or adult respiratory distress syndrome (ARDS) [1–3].

Surgical incisions by abolishing the integrity of the chest wall affect respiratory mechanics leading to impaired respiratory effort. Postoperative pain has been shown to be associated with decreased lung function by precluding deep inspirations. Furthermore, patients undergoing surgical procedures associated with opening the pleura will have increased rates of atelectasis, pleural effusions, and postoperative pain especially in the early postoperative period [4–6].

Cardiopulmonary bypass (CPB) can lead to the activation of different inflammatory and coagulation pathways and alters redox balance due to the passage of blood through the circuit (contact activation) and ischaemia and reperfusion injury [7–10].

Vascular endothelial cells (EC) dysfunction during CPB due to changes in blood flow patterns, shear stress, ischaemia, and reperfusion and circulating cytokines will result in the activation of multiple proinflammatory and proapoptosis pathways [11–14] while suppressing its ability to produce vasoprotective mediators [14–17]. EC activation is known to initiate leukocytes adhesion cascade by the expression of members of the selectin family which are responsible for the initial attachment of leukocytes from circulation. Transmigration of leukocyte through EC (crucial step for leukocytes recruitment to tissue) follows leukocytes attachment and is mediated by the upregulation of different adhesion molecules such as platelet endothelial cell adhesion molecule- (PECAM-) 1, ICAM-1, and very late antigen-4 (VLA-4) [18–21].

**Figure 1:**
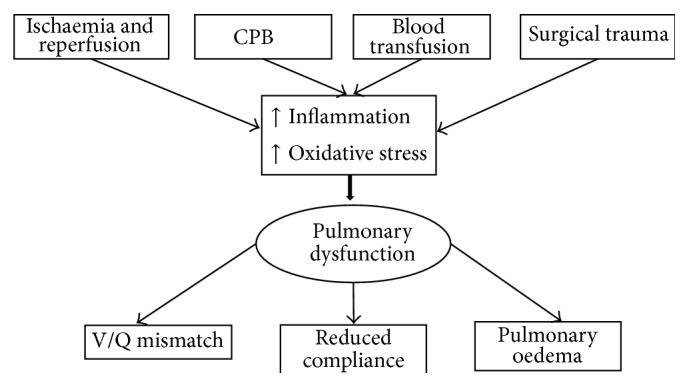
Mechanisms involved in pulmonary dysfunction during cardiac surgery and its consequences.

CPB is traditionally associated with inadequate lung perfusion as there is no flow to the pulmonary artery during periods of cross clamping and when the heart is not ejecting blood, and thus blood supply is limited to the bronchial arteries [22]. Pulmonary physiology alteration during CPB can result in disturbing the balance in the blood gas barrier due to the alteration of the different force affecting the parenchyma thus abolishing gas exchange by passive diffusion at the blood-gas barrier level and leading to ventilation/perfusion mismatch and impaired pulmonary compliance [23, 24]. Moreover, ischaemia during CPB is associated with reduced alveolar blood supply resulting in alveolar ischaemia and hypoxic pulmonary vasoconstriction [25]. Pulmonary vascular endothelial cells dysfunction and activated neutrophils sequestration into lungs parenchyma during period of reperfusion can increase lung tissue permeability and elevate vascular resistance and pulmonary surfactant changes leading to alveolar protein accumulation and pulmonary oedema and driving more ROS and cytokines production [26–28]. This will be manifested as abnormal gas exchange, poor lung mechanics, increased pulmonary shunt fraction and reduced functional residual capacity, and carbon monoxide transfer factor [28–30] (Figure 1).

Blood and blood products usage after surgery can be associated with the production of excessive amount of ROS and systemic and pulmonary inflammation. It has been previously shown that the duration of blood storage before transfusion can influence adverse effect associated with transfusion as there are an increased risk of respiratory insufficiency and the need for prolonged ventilation in patients receiving blood stored for longer than two weeks [10, 31–34].

Here we review the most commonly used strategies to minimise pulmonary dysfunction after cardiac surgery.

## 2. Pulmonary Protection Strategies

### 2.1. Pharmacological Interventions

The fact that inflammation and oxidative stress play a pivotal role in the development of pulmonary dysfunction resulted in multiple studies aimed toward modulating such events by the administration of vasodilators and antioxidant and anti-inflammatory medications in experimental animal models or patients undergoing cardiac surgery.

Prostacyclins can induce vascular smooth muscle cells relaxation by the release of intracellular adenosine 3′,5′ cyclic monophosphate (cAMP) resulting in pulmonary and systemic vasodilation [35–38]. Moreover, they can have inhibitory effect on platelet aggregation and leukocytes and monocytes activity [39]. The use of inhaled prostacyclins (Epoprostenol or Flolan) has been shown to decrease pulmonary arterial endothelial dysfunction induced by CPB in experimental studies [40–42]. Furthermore, when administered prior to CPB, they can have beneficial effect in the presence of pulmonary hypertension and may result in lower rate of reintubation in high risk postoperative cardiac patients [43, 44].

Phosphodiesterase inhibitors such as pentoxifylline (PTX) which is a known nonselective phosphodiesterase (PDE) inhibitor can result in elevated levels of intracellular cAMP and vasodilatation. Although PTX has been used classically for claudication symptoms in peripheral vascular disease [45], it has been shown to exert anti-inflammatory and antioxidative properties resulting in modulation of ALI [46–48]. More selective PDE inhibitors such as milrinone can be an advantageous therapeutic strategy for cardiac surgical patients with increased pulmonary vascular resistance (PVR) and right ventricular failure when nebulized and inhaled. It can cause selective pulmonary vasodilation and potentiate the vasodilation effects of inhaled prostacyclin [49, 50].

Nitric oxide (NO) is known to play a pivotal role in vascular endothelial cells homeostasis and regulation of oxidative stress and inflammatory responses [51]. Ischaemia and reperfusion injury during surgery is associated with significant loss in NO; thus NO preconditioning has been suggested to reduce perioperative pulmonary dysfunction and its sequels [52–54]. The protective effects may be due to reversal of postischemic lung hypoperfusion and reduction of lung neutrophil sequestration. The administration of NO in patients with severe left ventricular dysfunction can lead to pulmonary vasodilatation and may augment left ventricular filling [55–57]. The timing of administration and/or concentration of inhaled NO during ischaemia or reperfusion periods is a very important determinant of its effect as NO is toxic early in reperfusion, due to its interaction with superoxide which may lead to damage of alveolar type 2 cells [58, 59].

A large number of other drugs have been used with various degrees of success. Aprotinin (serine proteases) had been shown to reduce neutrophil elastase, malondialdehyde, and proinflammatory cytokines levels in bronchoalveolar lavage fluids of patients undergoing cardiac surgery [60]. The use of aprotinin can result in improving lung function and reducing reperfusion lung injury [61]. The administration of corticosteroids before CPB may reduce the activation of multiple proinflammatory mediators. The translation of proinflammatory mediator's changes into clinical outcomes remains controversial and most of the evidence in the literature originates from small RCTs or observational studies with biomarkers as primary end points [62, 63].

### 2.2. CPB Modification

Different strategies have been attempted over the years to minimise proinflammatory activation and oxidative stress when using CPB such as coating the circuit with biocompatible material (heparin, poly-2-methoxyethyl acrylate, synthetic protein, and phosphorylcholine), removal of leukocytes (by adding special filters to the CPB), ultrafiltration, and reduced haemodilution.

Heparin is thought to reduce the inflammatory responses linked to platelets and leukocytes by reducing the release of IL-6, IL-8, E-selectin, lactoferrin, myeloperoxidase, integrin, selectin, and platelet thromboglobulin and decreasing the production of oxygen free radicals [64–66]. It has been suggested that compared with conventional circuits (poly-2-methoxyethyl acrylate, synthetic protein, and phosphorylcholine), the heparin-coated circuit may improve lung compliance and pulmonary vascular resistance and thus reduce intrapulmonary shunt although intubation time and ICU stay were not affected [67, 68].

The use of leukocyte filtration mechanisms can modulate proinflammatory cytokines and oxidative stress [69–71]. A clinical study compared the effectiveness of leukocyte filter depletion with a common arterial filter in CABG patients who reported better oxygenation indices and less duration of postoperative mechanical ventilation in the leukocyte depletion filter group [71]. Another study suggested that leukocyte depletion filters preferentially remove activated leukocytes. Improvement in lung function was evident only in the early postoperative phase, but this did not lead to decreasing mortality or better clinical outcomes [72]. The use of ultrafiltration or modified ultrafiltration techniques at the end of surgery may reduce postoperative oedema specifically that of lungs resulting in better oxygenation and improved lung compliance postoperatively. Furthermore, ultrafiltration may remove proinflammatory mediators from the circulation such as IL-6 and IL-8 but it did not result in significant improvement of clinical outcomes [73]. Similarly, controlled haemodilution to regulate oncotic pressure can reduce priming volumes and result in better haemodynamic parameters such as vascular resistance and higher oxygen delivery and affect hospital stay significantly [74, 75].

Understanding problems associated with pulmonary ischaemia and reperfusion results in attempts to provide continuous pulmonary perfusion during CPB. Experimental animal models of pulmonary perfusion demonstrated reduced inflammatory and apoptosis pathways activation with such strategy [25, 76, 77]. Moreover, pulmonary perfusion was found to have favorable effect on lung compliance, oxygenation, and vascular resistance in patients undergoing CABG [78, 79]. Furthermore, pulmonary artery perfusion during CPB can be effective in reducing postoperative right ventricular dysfunction in high-risk patients undergoing LVAD placement [80]. The translation of such changes into better clinical outcomes remains unclear and may be restricted to selective group of patients as demonstrated by a recent trial in patients with COPD undergoing cardiac surgery using CPB where no significant protective effect on lungs was documented [81].

The deleterious effects of surface contact activation as discussed previously have led to the development of minimised cardiopulmonary bypass circuit (mini-CPB). This is characterised by reduced surface area and thus priming volume and prevention of air-blood contact. The utilisation of mini-CPB has been shown to be associated with attenuated production of proinflammatory cytokines and complement activation and blunted leukocytes activation compared to conventional circuit. Markers of oxidative stress tend to be reduced in patients undergoing surgery using mini-CPB compared to conventional circuit [82, 83]. Additionally, mini-CPB reduces organ damage and results in better postoperative gas exchange and lower lung injury scores [84, 85]. Unfortunately most of the clinical trials investigating the role of mini-CPB have evaluated diverse technologies of varying complexity and degree of miniaturisation, which would be expected to give rise to heterogeneity in findings.

### 2.3. Surgical Strategies

It has been suggested that eliminating the usual standard of no lung inflation during CPB by maintaining a degree of lung ventilation may be beneficial. The use of continuous positive airway pressure (CPAP) during CPB may result in less shunt and better gas exchange [86]; however, it seems that such effect is dependent on the airway pressure used. Using low frequency ventilation (LFV) along with CPAP during CPB to reduce post-CPB lung injury has been evaluated in an experimental pig model [87]. This study showed that the use of LFV is associated with significantly better pulmonary gas exchange, higher adenine nucleotide, lower LDH levels, and reduced histological damage in lung biopsies as well as lower DNA levels in bronchoalveolar lavage (BAL) compared to the collapsed lungs control group. However, a clinical study in patients undergoing cardiac surgery compared the effect of low volume ventilation to conventional strategy of no ventilation and demonstrated no significant changes in PVRI, PaO(2)/FiO(2) ratio, postoperative length of stay, and postoperative pulmonary complications [88]. Furthermore, a meta-analysis of 814 cases in 16 RCTs looking at three lung protective strategies in patients during CPB including CPAP, low-volume ventilation, and vital capacity manoeuvres during CPB showed that the effects of the designated techniques are probably short lived with a questionable impact on the long term clinical outcome of the treated patients [89].

Off pump coronary artery bypass (OPCAB) surgery seems to provide better lung protection by eliminating ischemia-reperfusion injury through maintaining lung ventilation and avoiding CPB. Many studies consistently reported better early and midterm outcomes in OPCAB when compared with conventional on-pump CABG: fewer respiratory complications, shorter intubation time and ITU stay, reduced incidence of pneumonia, and overall shorter hospital stay [90–92].

### 2.4. Physiotherapy

Preoperative prophylactic physiotherapy with inspiratory or expiratory muscle training can be used as a preventative measure for lung protection [93, 94]. Postoperative physiotherapy is used prophylactically in patients undergoing cardiac surgery. Different techniques can be utilised during this period to improve ventilation-perfusion inequalities, increase pulmonary compliance, and help reinflate collapsed alveoli.

These techniques include deep breathing exercises, slow maximal inspirations with an inspiratory hold, intermittent deep breathing exercises with and without the use of incentive spirometer, and deep breathing exercises with expiratory resistance [95–98].

### 2.5. Postoperative Noninvasive Ventilation (NIV)

NIV refers to the administration of ventilatory support without using an invasive artificial airway (endotracheal tube or tracheostomy tube). NIV exerts its main effects on the pulmonary and on the cardiovascular systems through the application of a positive end-expiratory pressure (PEEP); with or without a pressure support during inspiration, NIV restores lung volumes by opening atelectatic areas, increases alveolar ventilation, and reduces the work of breathing [99–101].

Continuous positive airway pressure (CPAP) aims to maintain a level of positive airway pressure in a spontaneously breathing patient. It is functionally similar to positive end-expiratory pressure (PEEP), except that PEEP is an applied pressure against exhalation and CPAP is a pressure applied by a constant flow. The ventilator does not cycle during CPAP, no additional pressure above the level of CPAP is provided, and patients must initiate all of their breaths. To avoid drying of the respiratory mucosa, there has been general agreement that the application of humidified CPAP helps to recruit the lungs by increasing functional residual capacity (FRC), increase the surface area of lung, decrease intrapulmonary shunt, and improve oxygenation [102–104].

Bilevel positive airway pressure (BLPAP) is a continuous positive airway pressure with pressure support breaths. It delivers a preset inspiratory positive airway pressure (IPAP) during inspiration and expiratory positive airway pressure (EPAP). BLPAP can be described as CPAP with a time-cycled or flow-cycled change of the applied pressure level [105]. BLPAP senses patients breathing efforts by monitoring air flow in the patient's circuit and adjusts its output by assisting inspiration. Therefore, its physiological effects can benefit the patient in both phases of respiration [106–108]. BLPAP application can only be commenced on conscious, cooperative, and hemodynamically stable patients who can breathe spontaneously, have an adequate gag and cough reflex, and are able to remove the mask when required. Several studies have demonstrated beneficial effects of BLPAP in reducing pulmonary complications and overall length of hospital stay after cardiac surgery [109–111]. Furthermore, the prophylactic use of BLPAP after early extubation has been shown to be safe and effective [111, 112]. A better tolerance was noted when BLPAP settings were commenced on low level and gradually adjusted to achieve the therapeutic target. Radiological improvement of atelectasis after cardiac surgery has been achieved on maintaining 8–10 mL/kg of tidal volume with BLPAP [113].

## 3. Conclusions

Pulmonary dysfunction is one of the most common and serious complications after cardiac surgery and can significantly impact on patient outcomes and health economics. The mechanisms involved in the development of pulmonary dysfunction are multifactorial and are related to the activation of different inflammatory and oxidative stress pathways. Clinical manifestation varies from mild atelectasis to severe respiratory failure. Managing pulmonary dysfunction postcardiac surgery is a multistep process that starts before surgery and continues during both the operative and postoperative phases. Pulmonary protection strategies have evolved over the years with various degrees of success. The main weakness of the majority of studies is often being observational in nature, small sample size, or being concentrated on a single intervention. Managing pulmonary dysfunction needs to be a multistep process involving more than one modality for each step of the surgical pathway. A better understanding of available modalities and/or combinations will result in the development of customised strategies for the different cohorts of patients. This in turn will help reduce pulmonary dysfunction and hence improve early outcome and costs after cardiac surgery.
